# Supplementary material for: Mapping expectancy-based appetitive placebo effects onto the brain in women
Source: Nat Commun. 2024 Jan 4;15:248. doi: 10.1038/s41467-023-44569-1 (PMC10764825; doi:10.1038/s41467-023-44569-1)
Supplement: Supplementary file 1 — supplementary information [file 41467_2023_44569_MOESM1_ESM.pdf]

1. Replication of the suggestion-based placebo intervention on hunger ratings in the fMRI sample

2. Additional analyses of dietary decision-making
   2.1. Relationship between the food stimulus value and calorie content of food
   2.2. Correlation of tastiness to caloric density
   2.3. Frequency of response categories during dietary decision-making
   2.4. Responses by tastiness and healthiness categories
   2.5. Regulatory success
   2.6. Healthy eating index scores (HEI-2015)

3. Additional brain imaging results
   3.1. Correlation of brain activation with the tastiness and healthiness at the time of food choice

4. Computational modeling
   4.1. Priors for parameters
   4.2. Parameter recovery

5. Brain mediators of placebo effects on hunger experiences
   5.1. Results: ROI-based single-level mediation analysis

6. Multi-Source-Interference-Task
   6.1. Behavioral results
   6.2. Whole brain results

7. Details of the placebo intervention

8. SI Tables 1 – 22
9. Supplementary References

1. Replication of the suggestion-based placebo intervention on hunger ratings in the fMRI sample

As shown in Supplementary Figure 1 below, placebo effects on hunger ratings were replicated in the smaller fMRI sample. In more detail, for the behavioral pilots, we observed a significantly greater increase in hunger from baseline to the end of the experiment for the increased- (mean hunger $\text{increase}_{\text{baseline to end}}$ = 0.96, sem = 1.16) than the decreased-hunger (mean hunger $\text{decrease}_{\text{baseline to end}}$ = 0.22, sem = 0.14) suggestion group ($t(113) = 3.49$, $p < 0.001$, Cohen's d = 0.61). We observed a similar difference for the smaller fMRI sample: increased-hunger suggestion group (mean hunger $\text{increase}_{\text{baseline to end}}$ = 0.62, sem = 0.27), in which the participants reported being significantly hungrier at the end of the experiment than at baseline and than participants of the decreased-hunger (mean hunger $\text{decrease}_{\text{baseline to end}}$ = 1.35, sem = 0.22) suggestion group ($t(55) = 2.11$, $p = 0.04$, Cohen's d = 0.56). The suggestion effect was not significantly different in magnitude between the pilot and fMRI samples, as indicated by a non-significant hunger suggestion $\text{group}_{\text{increased vs decreased}}$ by testing $\text{time}_{\text{baseline vs end of experiment}}$ by sample $_{\text{behavioral pilots vs fMRI}}$ interaction: $F(2,343) = 0.4$, $p = 0.67$).

We therefore pooled the two groups for comparisons of the decreased- and increased-hunger suggestion groups for behavioral and computational variables.

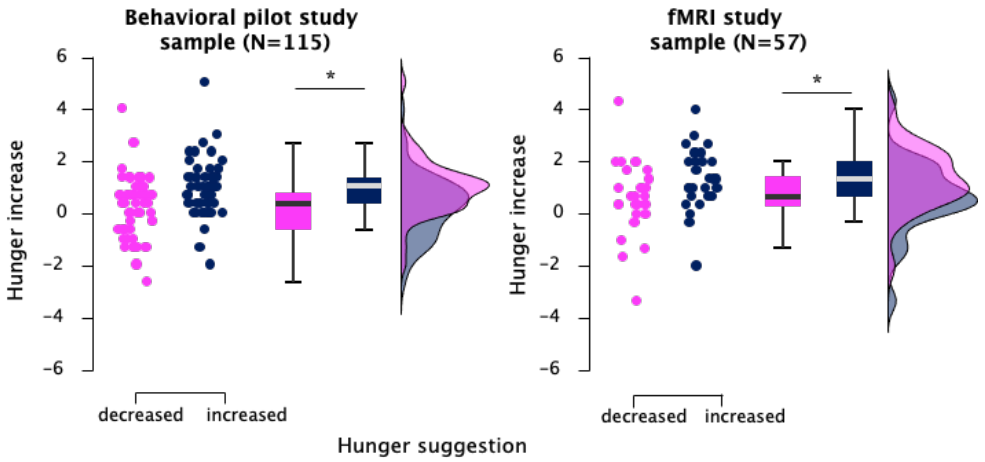

Figure S1. Placebo effects on hunger ratings for the pilot and fMRI samples. Raincloud plots for hunger ratings from baseline to the end of the experiment for the pilot behavioral sample (left side) and the fMRI sample (right side). Each dot corresponds to the difference in a participant's hunger rating from baseline to the end of the experiment, with positive values indicating an increase in hunger ratings. Distributions of the increase in hunger ratings are shown to the right of each plot. Boxplots show the 95% CI for both hunger-suggestion groups and samples. The black and gray lines in each boxplot indicate the median and the whiskers range from minimum and maximum values within 1.5 times the interquartile range. *$p < 0.05$. p-values were obtained with two-sampled, two-tailed t-tests. Source data are provided as a Source Data file.

2. Additional analysis of dietary decision-making

2.1. Relationship between the food stimulus value and calorie content of the food

Overall, we found that the participants did not assign significantly greater stimulus values to high-calorie food than low-calorie food, although there was a slight tendency ($t(254) = -1.92$, $p = 0.06$, paired-sample two-tailed t-test). We obtained the same result when comparing the stimulus values for low-calorie vs high-calorie food for all three groups: decreased-hunger suggestion group ($t(87)_D = -0.39$, $p = 0.70$, paired-sample two-tailed t-test), increased-hunger suggestion group ($t(83)_I = -1.60$, $p = 0.11$, paired-sample two-tailed t-test), and control group ($t(82)_C = -1.46$, $p = 0.15$, paired-sample two-tailed t-test).

The overall stronger correlation between stimulus value and high caloric food compared to the correlation between stimulus value and low-calorie food reported in the main text was driven by the increased-hunger suggestion group. Participants in this group showed a stronger correlation with high- (average Pearson's R = 0.21) than low- (average Pearson's R = -0.13) calorie foods (z = -2.16, p = 0.03, two-tailed r-to-z transformation). A similar pattern was observed for the control group (average Pearson's $R_{high} = 0.15$, $R_{low} = -0.13$, $z = -1.8$, $p = 0.07$, two-tailed r-to-z transformation) versus the decreased-hunger suggestion group (average Pearson's $R_{high} = 0.14$, $R_{low} = -0.11$, $z = -1.62$, $p = 0.11$, two-tailed r-to-z transformation. The differences between groups for the SV correlation were not significant for low-calorie or high-calorie food items. However, LME analysis (*Correlation Coefficient ~ 1 + group + calorie_level + group*calorie_level + (1 | sub_ID)*) showed a main effect of calorie level on the correlation coefficient ($\beta_{calorie\ level} = 0.14 \pm 0.01$, $t(506) = 16.41$, $p < 0.001$, 95% CI [0.13 – 0.16]) and a significant group by calorie level interaction ($\beta_{group*calorie\ level} = 0.02 \pm 0.01$, $t(506) = 2.04$, $p = 0.04$, 95% CI [8.40e-4 – 0.04]).

2.2. Correlation of tastiness ratings with caloric density

To assess whether the subjective taste ratings correlated with the calorie content of the food items, the calorie density (i.e., calories per gram) was correlated for each participant to the participant's tastiness ratings. Individual Pearson's correlation coefficients were then compared between the increased- and decreased-hunger suggestion groups, and the control group. Tastiness and calorie density did not correlate in the whole sample ($t(254) = -0.38$, p =

0.71, one-sample t-test), neither in the decreased- ($t(87)_D = -1.57$, p = 0.12), control- ($t(82)_C = 1.65$, p = 0.10) nor increased- ($t(83)_I = -0.44$, p = 0.66, one-sample t-test) hunger suggestion groups. Furthermore, there was no difference in the correlation coefficient of tastiness to calorie density between the groups, the LME analysis did not reveal a group effect ($\beta_{group} = 0.01 \pm 0.01$, t(253) = 0.87, p = 0.39, 95% CI [-0.02 – 0.04]), and post-hoc t-tests comparing the groups 2-by-2 did not show any significant difference. This finding indicates that the participants of our sample did not consider high-calorie food to be tastier.

2.3. Frequency of response categories during dietary decision-making

For each food, participants had to rate how much they wanted to eat the food at the end of the experiment using a 4-point Likert scale with four possible response types: strong no, no, yes, and strong yes. The occurrence of each response type (strong no, no, yes, strong yes) was calculated as the average percentage for a specific type of response:

$$x = \frac{\sum N_{response\ per\ type}}{N_{trial}} * 100$$

A two-factor LME analysis (group coded -1, 0, 1 and response type coded -2, -1, 0, 1, 2) revealed a main effect of response type ($\beta_{response\ type} = -3.36 \pm 0.32$, t(1271) = -10.47, p = < 0.001, 95% CI [-3.99 – -2.73]), with all participants responding more often with a strong no than any other response and a significant group by response type interaction ($\beta_{group*response\ type} = 1.35 \pm 0.39$, t(1271) = 3.45, p < 0.001, 95% CI [0.58 – 2.11]). Post-hoc t-tests results showed decreased hunger suggestion group to have significantly more “strong no” responses than the increased-hunger suggestion group (t(170) = 2.09, p = 0.04, Cohen’s d = 0.32, two-sample, two-tailed t-test, Figure S2). On the contrary, the increased-hunger suggestion group, as well as the control group, responded significantly more often with a “strong yes” than the decreased-hunger suggestion group (increased vs decreased: t (170) = -2.63, p = 0.01, Cohen’s d = -0.40; control vs decreased: t (169) = 2.61, p = 0.01, Cohen’s d = 0.40; two-sample, two-tailed t-tests). No difference was found for the frequency of “no” or “yes” responses between groups.

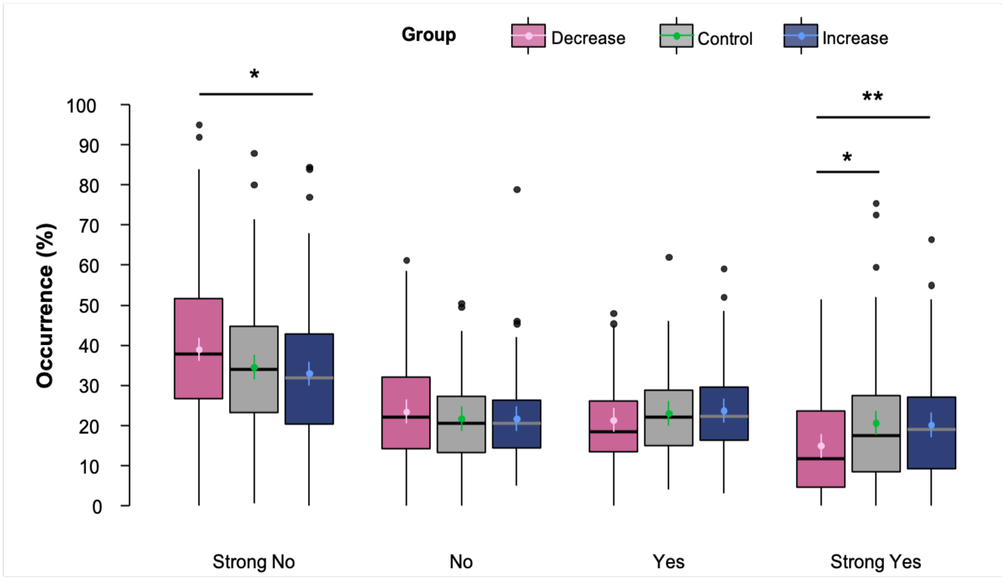

Figure S2. Average percentage of each type of response during the dietary decision-making task for the decreased- (pink bars, N = 88), increased- (dark blue bars, N = 84) hunger suggestion groups, and control group (gray bars, N = 83). Boxplots show 95% CI with black lines indicating the median, and whiskers range from minimum to maximum values within 1.5 times the interquartile range. Black dots indicate outlier values. **P < 0.01, *P < 0.05. P-values were obtained with two-sample, two-tailed t-tests. Source data and exact p-values are provided as a Source Data file.

2.4. Responses by tastiness and healthiness categories

Dietary decision-making trials were split into four categories based on the participants' tastiness and healthiness ratings: Healthy-Tasty, Healthy-Untasty, Unhealthy-Tasty, and Unhealthy-Untasty food choices.

To compare the three groups, we used an LME with the percentage of yes responses as the dependent variable and group (-1, 0, 1) and food category (1 for healthy and tasty, 2 for healthy but untasty, 3 for unhealthy but tasty, 4 for unhealthy and untasty) as fixed effects and found a main effect of category ($\beta_{\text{food category}}$ = -10.83 ± 0.80, t(1014) = -13.61, p < 0.001, 95% CI [-12.39 – -9.27]) but no effect of group nor an interaction between group and food category, indicating that all participants were more willing to consume tastier food items, regardless of group (Figure S3). However, participants in the increased-hunger suggestion group accepted healthy-tasty food more often (t(170) = -2.79, p = 0.006, Cohen's d = -0.43, two-sample two-tailed t-test) and rejected unhealthy-tasty food less often (t(170) = -3.34, p = 0.001, Cohen's d = -0.51, two-sample two-tailed t-test) than participants in the decreased-hunger suggestion group (Figure S3). The control group also chose healthy-tasty foods significantly more frequently than the decreased hunger group (t(169) = 3.24, p = 0.001,

Cohen's d = 0.50, two-sample two-tailed t-test). No significant differences between suggestion groups were observed for healthy-untasty or unhealthy-untasty foods.

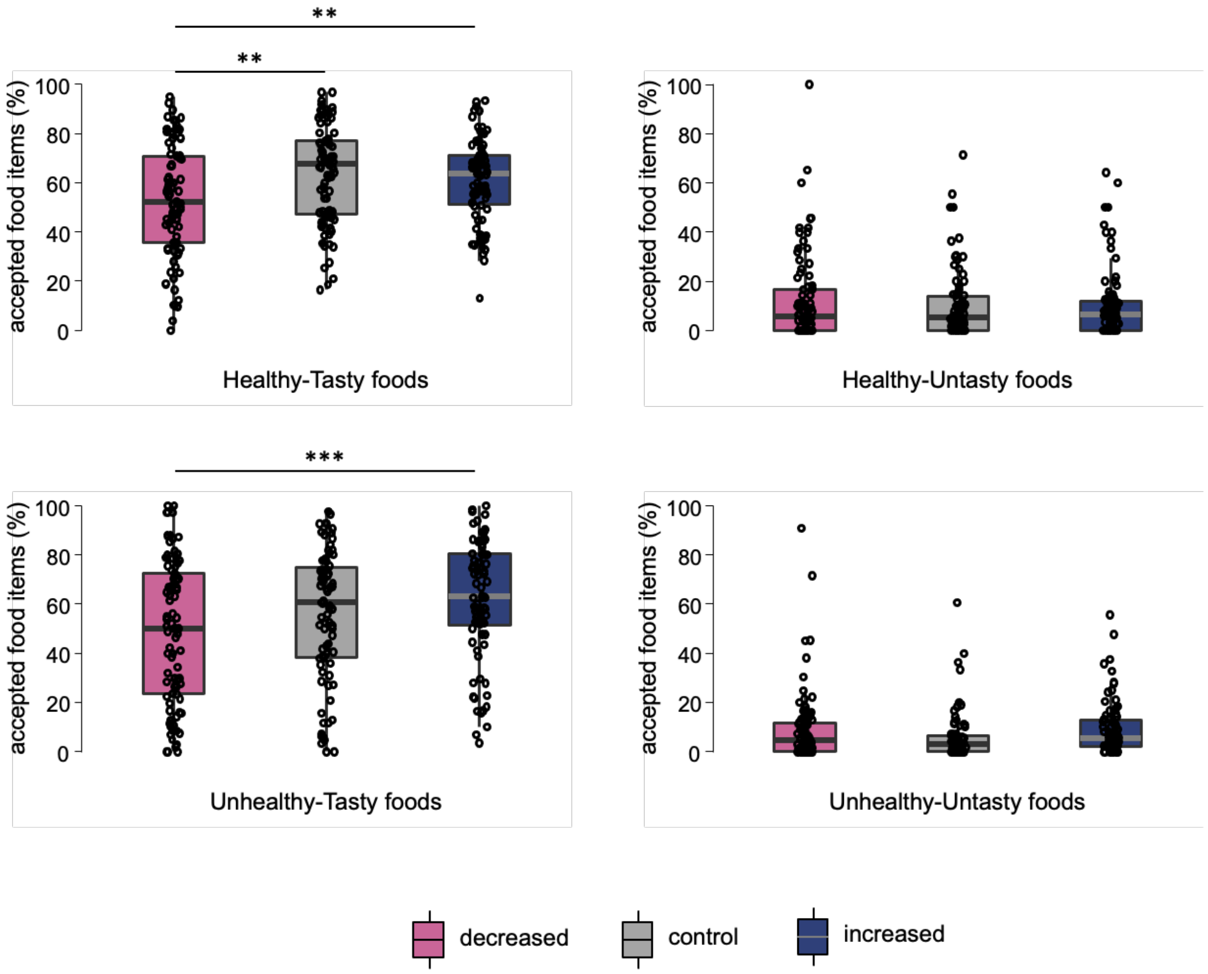

Figure S3. Average percentage of binned food acceptance (yes + strong yes responses) as a function of the healthiness and tastiness categories of food in each hunger suggestion group (N = 88 for decreased, N = 84 for increased, and N = 83 for control group). The boxplots show 95% CI with black and gray lines indicating the median, and whiskers range from minimum to maximum values within 1.5 times the interquartile range. Dots indicate individual participant values. ***P < 0.001, **P < 0.01. P-values were obtained by two-sample, two-tailed t-tests. Source data and exact p values are provided as a Source Data file.

2.5. Regulatory success

The regulatory success score (RSS) during dietary decision-making was defined as the sum of accepted healthy-untasty food (yes & strong yes responses) and rejected unhealthy-tasty food (no & strong no responses) over all healthy-untasty and unhealthy-tasty food items using the formula:

$$\mathrm{RSS} = \frac{\Sigma\ \mathrm{Yes\ healthy, untasty} + \mathrm{No\ unhealthy, tasty}}{\Sigma\ \mathrm{healthy, untasty\ food} + \ \mathrm{unhealthy, tasty\ food}}$$

A linear mixed-effects model was fitted to the regulatory success score with group as a predictor variable and BMI added as a regressor. The outcomes highlight a main effect of group (β_{group} = -0.05 ± 0.01, t(252) = -3.62, p < 0.001, 95% CI [-0.08 – -0.02]) and no effect of BMI.

After conducting a post-hoc t-test, there was a significant difference between the two hunger-suggestion groups (t(170) = 4.01, p < 0.001, Cohen's d = 0.61, two-sample two- tailed t-test; Figure S4), and between the decreased-hunger suggestion group and control group (t(169) = -2.40, p = 0.02, Cohen's d = -0.371, two-sample two- tailed t-test; Figure S4). Participants in the decreased-hunger suggestion group showed a greater RSS (mean RSS_D = 0.38 ± 0.02) than participants in the increased-hunger suggestion group (mean RSS_I = 0.26 ± 0.02) and those in the control group (mean RSS_C = 0.31 ± 0.02). Once again, the increased hunger group and the control group did not show any significant difference in the regulatory success score.

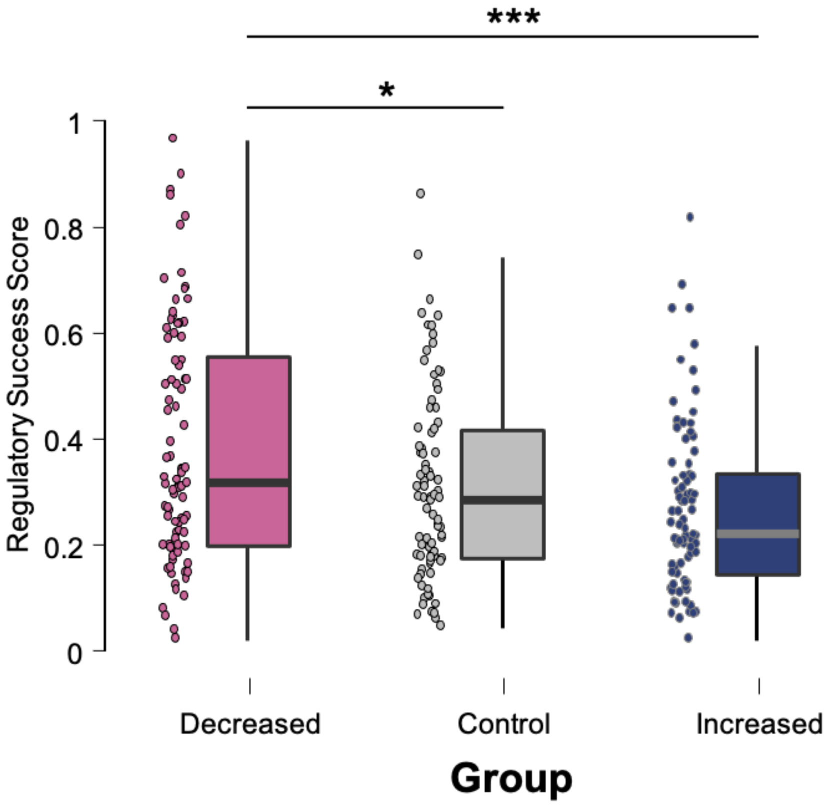

Figure S4. Boxplots displaying the 95% CI for the regulatory success score according to the suggestion group (decreased hunger (pink, N = 88) or increased hunger (dark blue, N = 84), and the control group (gray, N = 83)), with jitter elements showing dots for the individual score of each participant. The black and gray vertical lines indicate the median and the whiskers range from minimum to maximum values

within 1.5 times the interquartile range. Dots indicate individual participant values. *P < 0.05, ***P < 0.001. P-values were obtained with two-sample, two-tailed t-tests. Source data and exact p values are provided as a Source Data file.

2.6. Healthy eating index scores (HEI-2015)

A 24-hour recall questionnaire ascertained the complete dietary intake of the previous 24 hours for the control participants and those who underwent fMRI. This questionnaire involved obtaining a list of all food items eaten on the previous day by a trained dietitian (the 2nd author of the study). The interviewer probed for frequently forgotten foods, such as savory snacks, sugary beverages, candies, etc. Then, the time and name of the eating occasion (i.e., breakfast, lunch, dinner) and a detailed description of the type of food item, cooking method, and other relevant aspects were collected.

This information was then used to calculate the energy intake and the healthy eating index (HEI-2015) from the day before for each participant. The HEI-2015 scores were computed based on the Dietary Guidelines for Americans 2015-2020 (DGA, and following Krebs-Smith et al. 2018[1]). These guidelines consist of 13 components scored following adequacy- or moderation-based energy-adjusted intake cut-offs, (except for the fatty-acid components). Each component weights differently in the final scoring, which ranges between 0-100, where higher scores indicate a healthier dietary pattern. The components and their respective weights were: total fruits (5 points), whole fruits (5 points), total vegetables (5 points), greens and beans (5 points), whole grains (10 points), dairy (10 points), total protein foods (5 points), fatty acids (polyunsaturated fatty acid plus the monounsaturated fatty acid to saturated fatty acid ratio) (10 points), refined grains (10 points), sodium (10 points), added sugars (10 points), and saturated fats (10 points). Refined grains, sodium, added sugars, and saturated fats, as components to be consumed in moderation, were reversely scored[1] . A recall was also conducted one month after the experiment and the results were essentially the same, which indicates that this index is a stable measure of everyday diet across a period of one month.

As shown in SI table 16 HEI-2015 scores indicated that eating was comparable and not different between the two hunger-suggestion groups and the control group, and eating patterns were still at similar levels one month after the experiment for the two suggestion groups. Although this evidence provides an indirect indication of the status of the energy

stores on the day of the experiment, it provides some evidence that the eating habits of the participants did not differ the day before the experiment.

3. Additional brain imaging results during dietary decision-making

3.1. Brain activation in correlation with tastiness and healthiness

To localize where in the brain the tastiness and healthiness attributes were encoded and whether such encoding differed between the two hunger-suggestion groups, we fitted two general linear models (GLMs) to BOLD timeseries with the following regressors: an onset regressor at the time of choice (duration: reaction time) parametrically moderated by tastiness (GLM1) or healthiness (GLM2). Both GLMs also included onset regressors for missed trials (boxcar durations of 3s) and the six realignment parameters as regressors of non-interest to correct for head movements. Individual beta estimates for each regressor were then fitted into a second-level random effects analysis using one-sample t-tests for the entire sample (N=57) and two-sample t-tests to compare the decreased- (N=28) to increased- (N=29) hunger suggestion group.

Tastiness at the time of food choice activated the ventromedial prefrontal cortex (vmPFC), posterior cingulate cortex (PCC), precuneus, frontal eye fields, and dorsolateral and dorsomedial prefrontal cortices ($p_{FWE} = 0.05$, family-wise error corrected for the whole brain based on peak height; Figure S6a, SI Table 10). No differences were observed between groups, even with more lenient uncorrected thresholds ($p < 0.001$). Healthiness at the time of food choice was encoded in the bilateral amygdala ($p_{FWE} = 0.05$, family-wise error corrected based on peak height, Figure S6b, SI Table 11). At a lower uncorrected threshold, additional activation was found in the medial prefrontal cortex ($p < 0.001$ uncorrected; Figure S6b, SI Table 5). No differences in brain responses to healthiness at time of food choice were found between hunger-suggestion groups. Interestingly, conjunction maps showed the mPFC to correlate with both attributes, but positively with tastiness and negatively with healthiness. This finding suggests that this region integrates the weights of these two attributes differently.

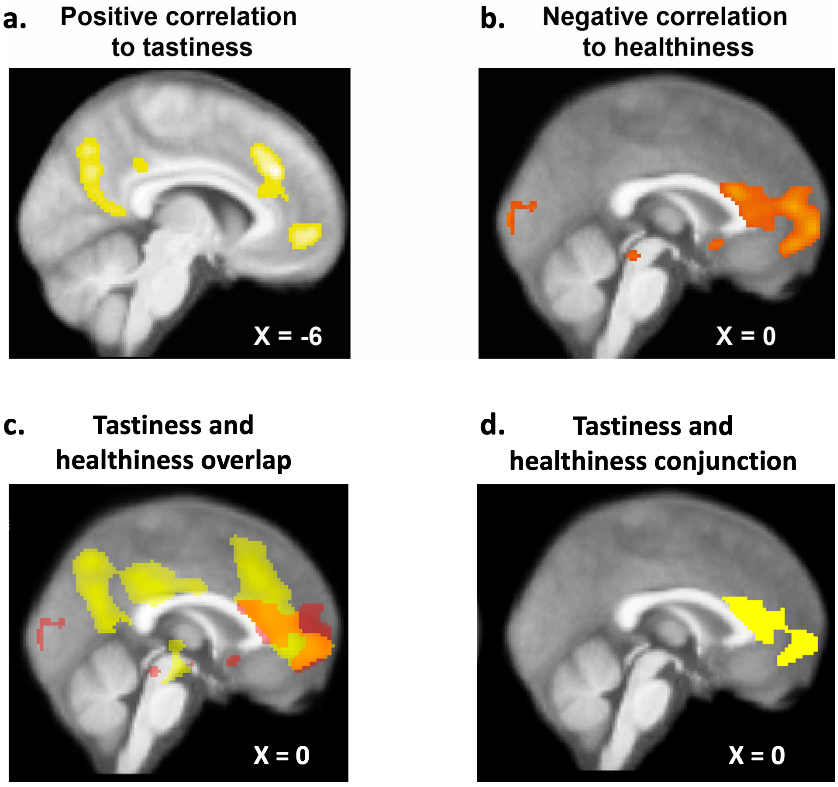

Figure S5. Brain activation at the time of food choice in response to (a) the tastiness and (b) healthiness of food. Statistical parametric maps (SPMs) for tastiness are displayed at $p_{FWE} < 0.05$ corrected for multiple comparisons using family-wise error whole brain corrected the on voxel level. The SPMs for healthiness are displayed at $p < 0.001$ uncorrected with an extent threshold of $k = 45$ for healthiness. Voxels in yellow and red are superimposed on the average anatomical image. **(c)** Conjunction map of tastiness (yellow) and healthiness (red) SPMs displayed at $p < 0.001$, uncorrected. **(d)** SPMs for common regions activated in response to tastiness and healthiness displayed at $p < 0.001$, uncorrected.

4. Computational modeling

4.1. Priors for each parameter at each level of the hierarchy

We used a hierarchical Bayesian model with three levels of hierarchy: the trial level (t), the participant level (p), and the condition (group) level (C) (Figure S6). The model estimated the most optimal parameters for hidden latent variables of the decision process at each level of the hierarchy, from the lowest trial-by-trial level to the participant and highest condition (group) level. In practice, this involved the model estimating a parameter for the threshold boundary separation (α_{cp}), non-decision-time (θ_{cp}), starting bias ($bias_{cp}$), and drift weights for tastiness ($w_{tastecp}$) and healthiness ($w_{healthcp}$). The model was separately fitted to the three experimental groups: the decreased-hunger suggestion (N = 88), increased-hunger suggestion (N = 84), and behavioral control group (N = 83).

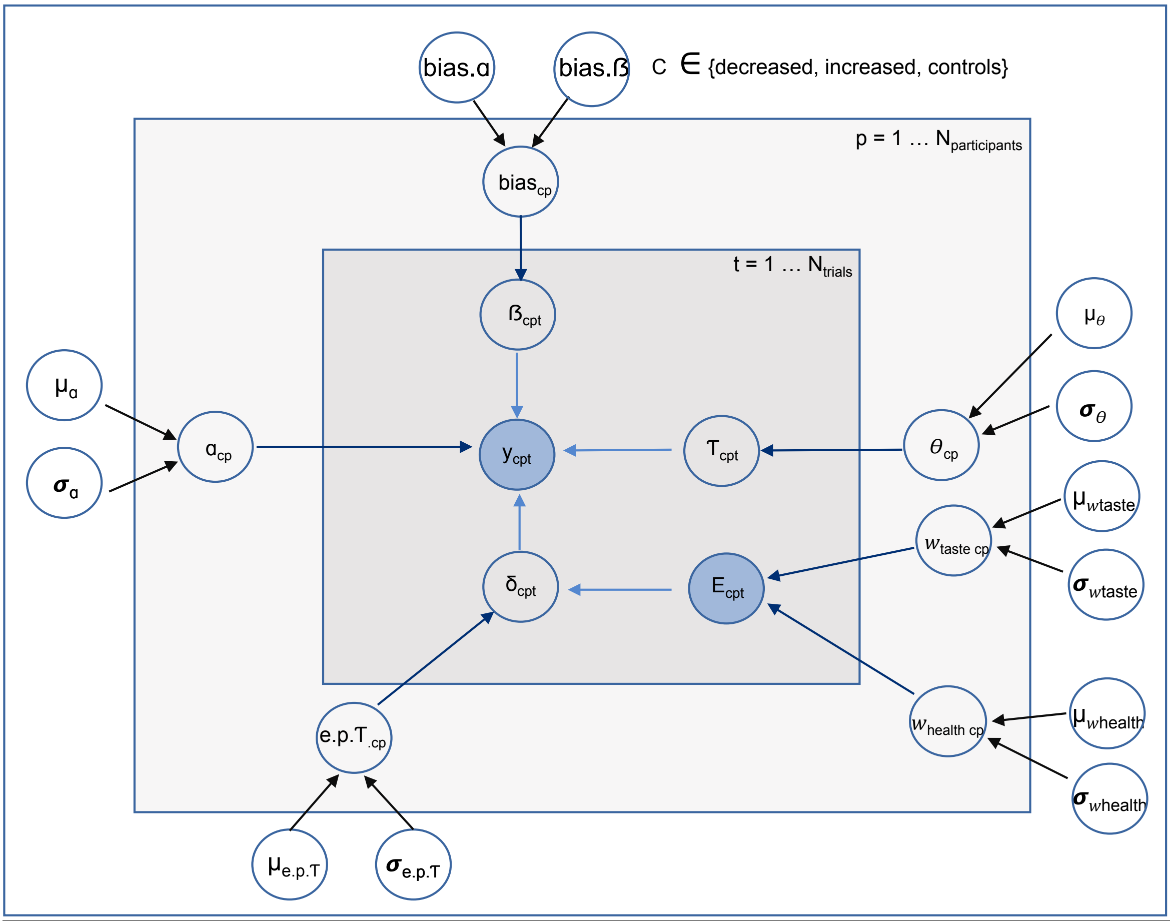

Figure S6. Scheme of the hierarchical Bayesian tDDM that was fitted to the observed choices and reaction times. The blue shapes denote the observed variables that varied on a trial-by-trial basis: the evidence, E_{cpt} (defined by equations 6, main text), and choice and reaction time data, y_{cpt} (see below). Hidden latent variables are shown as clear shapes at every level of the hierarchy.

4.1.1. Variables on a trial-by-trial level

The priors for predicted choice and reaction time data (y_{cpt}) were drawn from each trial from a univariate Wiener distribution using the formula:

$$y_{cpt} \sim \text{dwieners}(\alpha_t = 2\ ,\ T_{cpt},\ \beta_{cpt},\ \delta_{cpt}\ ,\ \alpha_{cp})$$

Priors for hidden latent variables on the trial-by-trial level were defined by:

- Non-decision time variable $T_{cpt} = \theta_{cp}$.
- Initial starting bias $\beta_{cpt} = \text{bias}_{cp}$.
- drift rate $\delta_{cpt} \sim \text{dnorm}(E_{cpt}, \text{e.p. } T_{cp})$.

4.1.2. Variables on the participant level:

The priors for the hidden parameters θ_{cp}, $bias_{cp}$, and error term e.p. $Ƭ_{cp}$ of the drift rate were defined at the participant level by:

- θ_{cp} ~ dnorm(μ_{θ}, $\boldsymbol{\sigma}_{\theta}$)
- e.p. $Ƭ_{cp}$ ~ dgamma(e.sG, e.RG)T(0.001, 20)
- $bias_{cp}$ ~ dbeta(bias. α, bias. β)T(0.01, 0.99)

The priors for the drift weights of tastiness and healthiness were drawn from a univariate normal distribution with the mean μ and precision $\boldsymbol{\sigma}$ using the formula:

- $w_{tastecp}$ ~ dnorm(μ_{wtaste}, $\boldsymbol{\sigma}_{wtaste}$)
- $w_{healthcp}$ ~ dnorm($\mu_{whealth}$, $\boldsymbol{\sigma}_{whealth}$)

The prior for the participant specific threshold boundary separation was defined by:

- α_{cp} ~ dnorm(μ_{α}, $\boldsymbol{\sigma}_{\alpha}$)

4.1.3. Variables at the condition (suggestion group) level:

At the highest level of the hierarchy, the condition (group) level, the model assumed flat uniform priors for latent variables, which are also called hyper-condition parameters and corresponded to the mean μ and precision $\boldsymbol{\sigma}$ of the prior distributions for some of the participant level parameters:

- μ_{wtaste} = unif(-5, 5); $\boldsymbol{\sigma}_{wtaste}$=gamma(1, 0.1)
- $\mu_{whealth}$ = unif(-5, 5); $\boldsymbol{\sigma}_{whealth}$=gamma(1, 0.1)
- μ_{α} = unif(0.001, 2); $\boldsymbol{\sigma}_{\alpha}$=gamma(1, 0.1)
- μ_{θ} = unif(0, 10); $\boldsymbol{\sigma}_{\theta}$=gamma(1, 0.1)

- e.SG = pow(e.m.,2) / pow(e.d.,2)
- e.RG = e.m. / pow(E;d.,2)
- e.m. ~ gamma(1, 0.2)T(0.001, 20)
- e.d. ~ gamma(1, 0.5)T(0.001, 20)

- bias. α = μ_{bias} * bias.kappa
- bias. ß = (1 – μ_{bias})* bias.kappa
- μ_{bias} = beta(2, 2) T(0.01, 0.99)
- bias.kappa ~ gamma(1, 0.5)

4.2. Parameter recovery

To determine if parameters obtained from the winning 2 weights DDM were identifiable, a parameter recovery was conducted.

To this aim new data was simulated for choices and reaction times using the DDM model and the RWiener package with the optimal parameter values and observed tastiness and healthiness ratings for the 255 participants. The total trial numbers were determined by each participant's number of observed food choices. The simulated data was then fitted by the 2 weights DDM using the same estimation method in JAGS as for the generating parameters, which gave a new set of recovered parameters. Both sets of parameters were then compared using Pearson's correlations. Parameter recovery was good (Figure S7 and SI Table 19). The generating and recovered parameters correlated significantly for the parameters of interest (SI Table 19).

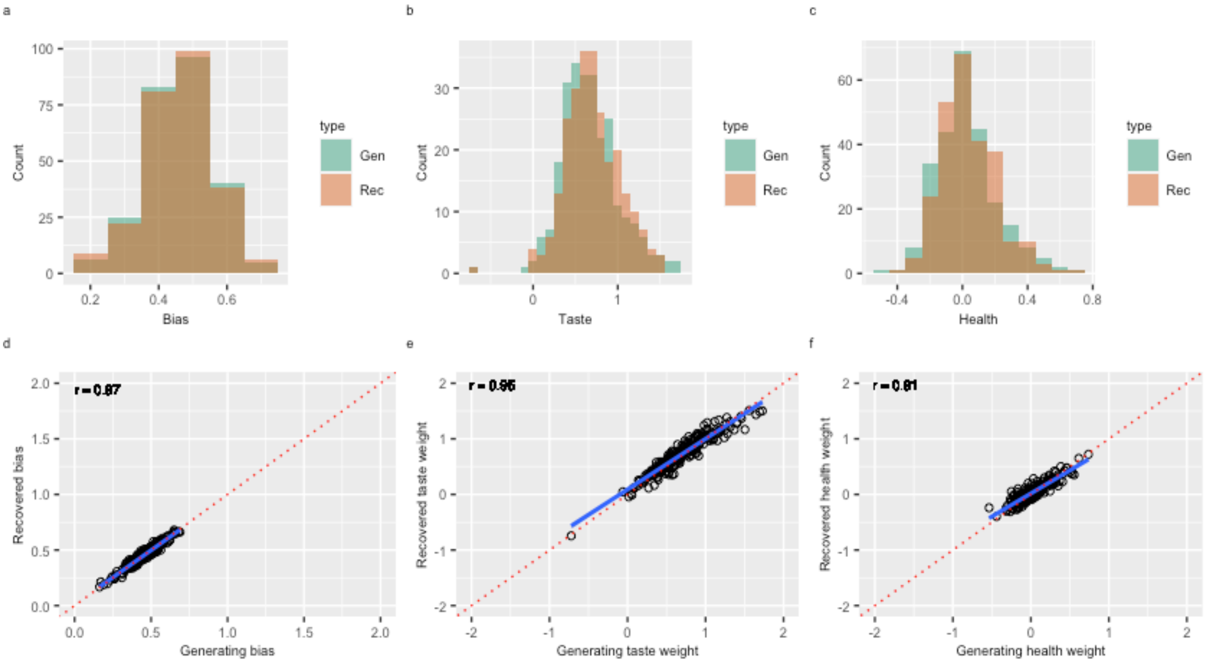

Figure S7. Parameter recovery for N = 255 participants. The histograms show the distribution of **(a)** the starting point bias, **(b)** the taste and **(c)** health drift weights for N = 255 participants. Panels below show the correlations (Pearson correlation coefficient) between the observed (generating) and recovered parameters for (**d)** the starting point bias, **(e)** taste and **(f)** health drift weight. The red dotted line indicates the x = y identity line. Source data are provided as a Source Data file.

5. Brain mediators of placebo effects on hunger experiences

The serial path regressions for the dlPFC mediation model showed a significant path a effect (beta = 0.49, se = 0.22, p = 0.02), which reflected the univariate effect of the hunger-suggestion group (increased > decreased hunger suggestion) on dlPFC activation at the time of food choice. After controlling for the path a effect, dlPFC activation significantly predicted the change in hunger from baseline to the end of the experiment (path b: beta = 0.32, se = 0.11, p < 0.01). Finally, mediation of the suggestion on hunger was significant (path a*b beta = 0.15, se = 0.09, p < 0.01), with a non-significant total effect of hunger suggestion on the hunger ratings after controlling for the mediator (path c': beta = 0.21, se =0.17, p = 0.26). These findings suggest that the group difference between increased- and decreased-hunger suggestions on hunger ratings can be formally explained by the activation of interference-resolution regions located in the dlPFC at the time of making the food choice.

6. Multi-Source-Interference task (MSIT)

To localize brain activation related to cognitive regulation, such as attentional filtering of task-relevant information, the participants of the fMRI experiment performed two sessions of the multi-source interference task (MSIT; SI Figure 8). The task design followed a previously reported procedure[2–4]. Briefly, the goal of the task was to select the digit 1, 2, or 3 as quickly and accurately as possible by pressing the corresponding response button (i.e., index finger for '1', middle finger for '2', and ring finger for '3'). During congruent control trials, the target number matched its position within zero distractor digits on the computer screen (e.g., 1 0 0 or 0 2 0 or 0 0 3). On the contrary, during incongruent interference trials, the target digit never matched its position within the non-zero distractor digits (e.g., 2 1 2). This condition created a Stroop effect and required allocation of attentional resources to inhibit task-irrelevant information and filter task-relevant information to provide a correct response. Before performing this task within the fMRI scanner, all participants trained for a full session outside the scanner.

6.1. Behavioral Results

Four participants were excluded from the behavioral analyses because they misunderstood the task instructions. These four participants reported the place of the target number for the incongruent trials. For example, they pressed the third response button for a "1 1 2" trial, whereas the correct answer was pressing the second button. Two of them performed the training task perfectly but failed during the MRI session despite the instructions being repeated before each session. For the other two participants, performance during the incongruent trials was already close to zero during the training session. For these two participants, it appears that the instructions were not well understood and that the experimenter remained unaware of this during the training session.

For the remaining participants, the effect of interference by task-irrelevant information was measured using two linear-mixed-effect models (LMEs) applied to the two behavioral measurements of interest: performance (i.e., % correct responses) and reaction time (RT, in seconds). In accordance with the literature, participants showed a significantly highly number of errors during the incongruent than congruent trials (SI Table 14), which was indicated by a main effect of trial type (congruent or incongruent) on both performance (β =

5.90, t(199) = 5.51, $p < 0.001$) and reaction time (β = -0.18, t(199) = -13.01, $p < 0.001$). For both measurements, there was no significant effect of group (performance: β = -0.91, t(199) = -0.40, p = 0.69, RT: β = 0.002, t(199) = 0.08, p = 0.93) or interaction of hunger suggestion group by type of MSIT trial (performance: β = -0.60, t(199) = -0.75, p = 0.46; reaction times: β = -0.001, t(199) = -0.23, p = 0.82). Furthermore, we observed a learning effect, as the difference in RT (ΔRT = RT incongruent trials – RT congruent trials) was significantly higher in session 1 than session 2 (t(49) = 3.65, $p < 0.001$, paired-sample two-tailed t-test). Neither performance nor RT differed between hunger suggestion groups (SI Table 20).

6.2. MSIT Brain Imaging Results

A multilevel general linear model contained two onset regressors for the congruent and incongruent trial blocks and six realignment parameters as covariates of non-interest to control for head movement. Individual beta estimates for the contrast between task condition (congruent versus incongruent) were then fitted into a second-level random effects analysis using one-sample t-tests to localize brain responses associated with interference resolution (congruent < incongruent) and the opposite effect (congruent > incongruent).

Whole brain activation for interference resolution (incongruent > congruent) involved the dorsal anterior cingulate cortex (dACC), dorsolateral prefrontal cortex (dlPFC), parietal cortex, and anterior insula, which were more highly activated in the incongruent than congruent trials ($p_{FWE} < 0.05$, family-wise error corrected based on peak height, Figure S8, SI Table 21). In contrast, stronger activation of the brain's reward and valuation system was found in the congruent than incongruent trials. This brain activation involved the vmPFC, posterior cingulate cortex, ventral striatum, hippocampus, and posterior insula (pFWE < 0.05, family-wise error corrected based on peak height, Figure S8, SI Table 22). No significant differences were found for the congruent versus incongruent contrast between the hunger suggestion groups.

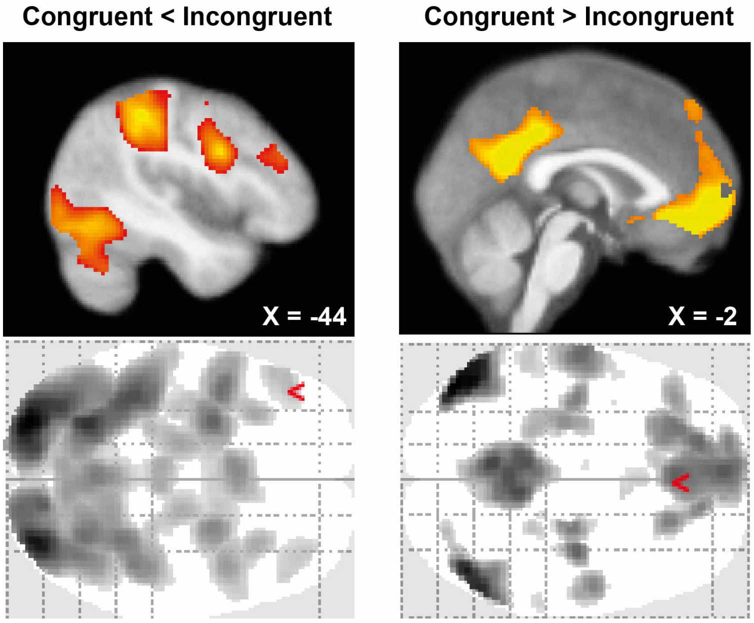

Figure S8. Brain activation during the MSIT task for interference resolution (congruent < incongruent trials) and control (congruent > incongruent trials). SPMs are displayed at $p_{FWE} < 0.05$, family-wise error whole brain corrected based on peak height. Significant voxels in red and yellow are superimposed on the average anatomical image.

7. Details of the placebo intervention

<u>Information about the drink given to the participants assigned to the decreased-hunger suggestion group:</u>

"Before starting the computer/fMRI task, we will ask you to drink a glass of this refreshing, non-carbonated, and neutral-flavored drink, which will help you to focus better on the exercises that you will perform later on the computer/during fMRI. This drink has exceptional nutritional benefits, such as the presence of amino acids, carbohydrates, and traces of elements that are essential for health and brain functioning. Specifically, it has been enriched with riboflavin, also known as vitamin B2, which acts positively on leptin secretion. Leptin is a natural hormone produced by your body that helps to burn fat and reduce food cravings. One glass of this drink is an excellent source of chromium, a formidable anti-sugar element that acts on the metabolism of carbohydrates. It is an insulin cofactor that promotes glucose absorption. Chromium deficiency can cause sugar cravings, with signs of hypoglycemia. Moreover, this water contains vitamins B6, B12, and E, stimulating the development of neural tissue and cell formation, which also act positively on the activity of the neurotransmitter serotonin, known as a well-being hormone involved in

mood and concentration. In addition, this water is enriched with mineral salts, such as magnesium and iron, which are essential in case of anemia and a valuable ally for women's health because they provide oxygen to the cells and reduce the feeling of fatigue. This drink is an excellent source of extra food during sports and intellectual endeavors. A 25-cl glass covers all the nutritional needs of the next two hours. This drink is natural, gluten-free, and packaged in a protected environment. You will have five minutes to drink a glass of the water. Thereafter, you should wait a few minutes to allow time for the active substance to deliver their effect. We will then ask you to perform a food-choice task."

<u>Information about the drink provided to participants in the increased-hunger suggestion group:</u>

"Before starting the experiment, we will ask you to drink a glass of this refreshing, non-carbonated, and neutral-flavored drink, which will help you to focus better on the exercises that you will perform later on the computer/during fMRI. This drink has exceptional nutritional benefits, such as the presence of amino acids, carbohydrates, and traces of elements that are essential for health and brain functioning. Specifically, it has been enriched with zinc, which promotes digestion, fights against heartburn, and stimulates the taste buds. It also brings together the benefits of medicinal plants such as St. John's wort, known for its stimulating effect on hunger. Its action is based on its ability to send a hunger message to the brain. Several studies have shown that this medicinal plant acts positively on the activity of the neurotransmitter serotonin, known as a well-being hormone involved in mood and concentration. In addition, this water is also enriched with mineral salts, such as magnesium and iron, essential in cases of anemia and a valuable ally for women's health because they provide oxygen to the cells and reduce the feeling of fatigue. This drink is natural, gluten-free, and packaged in a protected environment. You will have five minutes to drink a glass of the water. Thereafter, you should wait a few minutes to allow time for the active substance to deliver their effect. We will then ask you to perform a food-choice task.

8. Supplementary tables

SI Table 1. Positive moderation of brain activation by prognostic expectations at the time of food choice in the decreased-hunger suggestion group, N =28

Region	Brodmann Area	Side	Size	x	y	z	Peak z-score
IpS	39	L	63	-28	-80	38	4.28
mPFC*	10	R	355	2	58	22	3.98
AG	39	R	141	54	-66	24	3.71
dPCC	31	R	53	2	-56	34	3.48

IpS – intraparietal sulcus; AG – angular gyrus; mPFC – medial prefrontal cortex; dPCC – dorsal posterior cingulate cortex. Values are reported at p < 0.001, uncorrected, using an extend threshold of k = 50. *Regions that survived family-wise error correction based on the cluster level.

SI Table 2. Firstlevel results from a multilevel general linear model of stimulus value measured in the decreased hunger suggestion participants (N=88).

	intercept	TR	HR	trial	TRxHR	trialxTR	trialxHR
Coefficient	-0.69	0.54	-0.01	-3.76e-4	0.02	-2.81e-5	3.26e-4
STE	0.06	0.03	0.02	1.71e-4	0.01	1.23e-4	1.21e-4
t	-10.96	16.42	-0.37	-2.19	2.30	-0.23	2.69
Z	-8.21	8.21	-0.37	-2.15	2.26	-0.23	2.62
p	2.22e-16	2.22e-16	0.71	0.03	0.02	0.82	0.01

TR – Taste Ratings; HR – Health Ratings; STE – Standard Error. Source data are provided as a Source Data file.

SI Table 3. Firstlevel results from a multilevel general linear model of stimulus value measured in the control participants (N=83).

	intercept	TR	HR	trial	TRxHR	trialxTR	trialxHR
Coefficient	-0.67	0.67	0.02	1.17e-4	0.03	-8.88e-5	3.41e-4
STE	0.05	0.03	0.02	1.91e-4	0.01	1.38e-4	1.33e-4
t	-12.38	20.86	0.69	0.61	3.23	-0.64	2.57
Z	-8.21	8.21	0.69	0.61	3.12	-0.64	2.51
p	2.22e-16	2.22e-16	0.49	0.54	1.79e-3	0.52	0.01

TR – Taste Ratings; HR – Health Ratings; STE – Standard Error. Source data are provided as a Source Data file.

SI Table 4. Firstlevel results from a multilevel general linear model of stimulus value measured in the increased hunger suggestion participants (N=84).

	intercept	TR	HR	trial	TRxHR	trialxTR	trialxHR
Coefficient	-0.50	0.67	-0.05	-6.06e-4	0.02	-2.15e-5	3.15e-4
STE	0.06	0.03	0.02	0.00	0.01	1.37e-4	1.34e-4
t	-9.05	24.64	-2.58	-2.67	2.85	-0.16	2.34
Z	-7.53	8.21	-2.52	-2.61	2.78	-0.16	2.29
p	5.17e-14	2.22e-16	0.01	0.01	0.01	0.88	0.02

TR – Taste Ratings; HR – Health Ratings; STE – Standard Error. Source data are provided as a Source Data file.

SI Table 5. Second level, random effects results using weighted two-sample, two-tailed t-tests to compare individual beta estimates from the first level between increased- and decreased-hunger suggestion groups.

	t	df	p	Lower 95% CI	Upper 95% CI
intercept	-2.28	170	0.024	-0.65	-0.05
TR	-2.81	170	0.005	-0.73	-0.13
HR	1.08	170	0.283	-0.14	0.46
trial	1.39	170	0.167	-0.09	0.51
TRxHR	-1.02	170	0.311	-0.45	0.15
trialxTR	-0.82	170	0.415	-0.42	0.18
trialxHR	0.85	170	0.396	-0.17	0.43

TR – Taste Ratings; HR – Health Ratings. Source data are provided as a Source Data file.

SI Table 6. Second level, random effects results using weighted two-sample, two-tailed t-tests to compare individual beta estimates from the first level between decreased-hunger suggestion group and control group.

	t	df	p	Lower 95% CI	Upper 95% CI
intercept	-2.20	169	0.841	-0.33	0.27
TR	-2.92	169	0.004	-0.75	-0.14
HR	1.09	169	0.277	-0.47	0.13
trial	1.59	169	0.114	-0.54	0.06
TRxHR	-1.20	169	0.232	-0.48	0.11
trialxTR	-0.75	169	0.456	-0.41	0.19
trialxHR	0.63	169	0.530	-0.20	0.40

TR – Taste Ratings; HR – Health Ratings. Source data are provided as a Source Data file.

SI Table 7. Second level, random effects results using weighted two-sample, two-tailed t-tests to compare individual beta estimates from the first level between increased-hunger suggestion group and control group

	t	df	p	Lower 95% CI	Upper 95% CI
intercept	-2.26	165	0.025	0.04	0.66
TR	-0.41	165	0.683	-0.37	0.24
HR	-2.20	165	0.029	-0.65	-0.03
trial	-2.69	165	0.008	-0.72	-0.11
TRxHR	-0.20	165	0.845	-0.33	0.27
trialxTR	-0.02	165	0.983	-0.30	0.31
trialxHR	0.19	165	0.848	-0.33	0.27

TR – Taste Ratings; HR – Health Ratings. Source data are provided as a Source Data file.

SI Table 8. Second-level, random effects ANOVA Summary of the effect of Group on beta estimates from the first level.

	df	*F*	p
intercept	2, 252	3.35	0.037
TR	2, 252	5.69	0.004
HR	2, 252	2.33	0.099
trial	2, 204	4.49	0.012
TRxHR	2, 212	0.92	0.399
trialxTR	2, 252	0.39	0.676
trialxHR	2, 252	0.38	0.686

TR – Taste Ratings; HR – Health Ratings. Source data are provided as a Source Data file.

SI Table 9. Differential brain activation in response to the stimulus value at the time of food choice for the contrast increased > decreased hunger suggestion, N = 57.

Region	Brodmann Area	Side	Size	x	y	z	Peak z-score
Insula*	13	L	4632	-30	-14	18	4.15
	13	R	271	38	-4	18	4.51
vmPFC*#	10/11		691	0	52	-12	4.42
Cerebellum*		R	2652	12	-46	16	4.26
Precuneus*	7	R	580	2	-42	62	3.76
NAcc	25		47	0	6	-6	3.60
PCC	31	L	115	-8	-32	52	3.45

vmPFC – ventromedial prefrontal cortex; NAcc – nucleus accumbens; PCC – posterior cingulate cortex. Values are reported for $p < 0.001$, uncorrected. Regions marked with an * survived a $p_{FWE} < 0.05$, family-wise error correction based on the cluster and voxel level. # seed region of interest for PPI analysis.

SI Table 10. Positive effect of tastiness at the time of food choice for all 57 participants.

Region	Brodmann Area	Side	Size	x	y	z	Peak z-score
IpS	39	L	1122	-34	-62	38	5.89
dACC	32	L	420	-6	34	32	5.77
dlPFC	9	L	816	-32	20	50	5.64
	46	L	133	-44	42	18	5.40
vmPFC	10	L	260	-6	52	-4	5.33
Precuneus	17/18	L	526	-6	-64	32	5.26
PCC	31	L	75	-4	-38	36	4.91

IpS – intraparietal sulcus; dACC – dorsal anterior cingulate cortex; dlPFC – dorsolateral prefrontal cortex; vmPFC – ventromedial prefrontal cortex; PCC – posterior cingulate cortex. Values were taken at a $p_{FWE} < 0.05$, family-wise error correction for multiple comparisons based on peak height.

SI Table 11. Negative effect of healthiness at the time of food choice for N = 57 participants.

Region	Brodmann Area	Side	Size	x	y	z	Peak z-score
V1*	18	L	4886	-16	-98	6	6.88
Amygdala*		L	308	-22	-8	-18	5.31
Parahippo*	37	R	438	26	-48	-14	4.85
vmPFC	10		2610	0	64	0	4.18
STG	22	R	112	60	-10	-12	3.98
vlPFC	47	L	194	-38	28	-4	3.97
PCC	23	L	76	-16	-50	6	3.79
NAcc	25	L	64	-2	14	-8	3.59
MFG	8	L	50	-16	28	38	3.53

V1 – primary visual cortex; Parahippo – parahippocampus; vmPFC – ventromedial prefrontal cortex; STG – superior temporal gyrus; vlPFC – ventrolateral prefrontal cortex; PCC – posterior cingulate cortex; NAcc – nucleus accumbens; MFG – medial forntal gyrus. Values are reported for $p < 0.001$, uncorrected, extent threshold k = 45, regions marked with an * survived family-wise error correction ($p_{FWE} < 0.05$) based on peak height. Note, no significant activation was found for positive correlations with healthiness, even at $p < 0.001$, uncorrected.

SI Table 12. Average beta estimates extracted at the time of food choice from regions activated during interference resolution trials of the MSIT on N = 57 participants.

	**Decreased**	**Increased**
dACC [-4, 10, 52]	3.55 ± 0.34	3.71 ± 0.22
Insula [32, 22, 4]	1.95 ± 0.20	2.38 ± 0.20
dlPFC [40, 42, 26]	1.04 ± 0.29	2.03 ± 0.35

Individual beta estimates were first extracted from ROIs at the time of choice during the dietary decision-making task. Error values correspond to the standard error of the mean. dACC – dorsal anterior cingulate cortex; dlPFC – dorsolateral prefrontal cortex. Source data are provided as a Source Data file.

SI Table 13a. Comparison of the posterior distribution means for each population level free parameter between the decreased (N = 88) and increased (N = 84) hunger suggestion groups

	MEAN($PD_{INCREASED} - PD_{DECREASED}$)	PP	95% HDI	H1
$W_{HEALTHINESS}$	0.06 ± 0.04*	0.95*	[-0.09 – 0.21]*	Decreased > Increased
$W_{TASTINESS}$	0.20 ± 0.05	0.99	[-0.03 – 0.42]	Decreased < Increased
θ	0.02 ± 0.04	0.67	[-0.14 – 0.18]	Decreased < Increased
BIAS	0.04 ± 0.03	0.98	[-0.12 – 0.17]	Decreased < Increased

PD – posterior distributions of each parameter for decreased and increased suggestion groups. PP – posterior probability that differences are > zero. w – drift weights, θ – non-decision time, BIAS – initial starting point bias. Note, the * indicates that for the healthiness drift weight the average PDs and the PP were calculated assuming ($d_{decreased} - d_{increased} > 0$).

SI Table 13b. Comparison of the posterior distribution means for each population level free parameter between the decreased hunger suggestion (N = 88) and control (N = 83) group

	MEAN($PD_{CONTROLS} - PD_{DECREASED}$)	PP	95% HDI	H1
W	–0.04 ± 0.4*	0.14	[-0.19 – 0.11]*	Decreased > Controls
$W_{TASTINESS}$	0.16 ± 0.06	0.99	[-0.54 – 0.15]	Decreased < Controls
θ	–0.21 ± 0.03	0	[-0.35 – -0.09]	Decreased < Controls
BIAS	0.05 ± 0.02	0.99	[-0.02 – 0.12]	Decreased < Controls

* indicates that for the healthiness drift weight the average PDs and the PP were calculated assuming ($d_{decreased} - d_{controls} > 0$)

SI Table 13c. Comparison of the posterior distribution means for each population level free parameter between the increased hunger suggestion (N = 84) and control (N = 83) group

	MEAN($PD_{INCREASED} - PD_{CONTROLS}$)	PP	95% HDI	H1
$W_{HEALTHINESS}$	0.10 ± 0.03*	0.99*	[-0.02 – 0.25]*	Controls > Increased
$W_{TASTINESS}$	0.04 ± 0.05	0.75	[-0.19 – 0.27]	Controls < Increased
θ	0.23 ± 0.03	1.00	[-0.12 – 0.34]	Controls < Increased
BIAS	–0.01 ± 0.02	0.32	[-0.07 – 0.07]	Controls < Increased

* indicates that for the healthiness drift weight the average PDs and the PP were calculated assuming ($d_{controls} - d_{increased} > 0$)

SI Table 14. Whole brain activation for the psychophysiological interaction with the vmPFC as a seed ROI (N = 57).

Region	Brodmann Area	Side	Size	x	y	z	Peak z-score
SMG	40	L	843	-56	-40	46	6.20
	40	R	374	56	-36	44	5.41
Insula	13	R	80	34	4	8	5.56
	13	L	199	-42	2	4	5.26
STG	22	R	66	64	-38	16	5.28
dlPFC	46	L	73	-50	42	8	5.02
Premotor/SMA	44/6	R	99	54	12	14	4.97
	6	R	52	-58	6	16	4.97
Fusiform	37	R	65	56	-56	-8	4.90
IpS	39/7	R	45	38	-44	42	4.86
Putamen		L	54	-24	-4	6	4.76

SMG – supramarginal gyrus; STG – superior temporal gyrus; dlPFC – dorsolateral prefrontal cortex; premotor – premotor cortex; SMA – supplementary motor area; IpS – intraparietal sulcus.
Values are taken at $p_{FWE} < 0.05$, family-wise error corrected based on peak height.

SI Table 15. Sociodemographic, body composition, and questionnaire data (N = 255).

	Decrease	Control	Increase
Age (years)	35.67 ± 1.43	32.34 ± 1.35	32.67 ± 1.42
Education	3.82 ± 0.06	3.74 ± 0.07	3.86 ± 0.04
Weight (kg)	63.77 ± 1.64	60.67 ± 1.33	63.34 ± 1.38
BMI (kg/m²)	23.37 ± 0.56	22.22 ± 0.41	23.13 ± 0.44
Body Fat (%)	28.95 ± 1.11	28.10 ± 2.50	27.93 ± 1.08
SCOFF	1.37 ± 0.27	1.25 ± 0.14	1.32 ± 0.15
BDI	4.53 ± 0.55	2.65 ± 0.29	3.88 ± 0.50
YFAS	0.82 ± 0.22	1.09 ± 0.21	0.86 ± 0.27
IPAQ-sf	1625.70 ± 181.94	1988.50 ± 150.16	1359.92 ± 196.28

Note, education was coded as 1 for high school undergraduate, 2 for high school graduate, 3 for two years of higher education (college), and 4 for more than two years of college. SCOFF, YFAS, and IPAQ average scores were calculated for the fMRI study participants only (N = 57). SCOFF – Sick, Control, One stone, Fat, Food; BDI – Beck's Depression Inventory, YFAS – Yale Food Addiction Scale, IPAQ-sf – International Physical Activity Questionnaire short form. Source data are provided as a Source Data file.

SI table 16. HEI scores for N = 57 participants of the fMRI experiment and N=54 participants of the control study.

	Hunger suggestion	Mean	SEM	Minimum	Maximum
Day before	Decreased	48	2.9	20	78
	Increased	45	3.1	17	80
	Controls	50	1.5	34	80
One month later	Decreased	52	3.8	21	80
	Increased	51	2.8	16	81

Note, healthy eating indexes scores were calculated following Krebs-Smith et al. (2018)[1]. A score of 100 indicates total correspondence of eating behavior with the 2015-2020 Dietary Guidelines for Americans. No differences between groups on the HEI-2015 scores were observed for eating on the day before the fMRI~~,~~ and one month later. SEM – standard error of the mean. Source data are provided as a Source Data file.

SI table 17. Food stimuli used during the dietary decision-making task for the behavioral pilot experiment (n=200 trials) and the fMRI experiment (n=152 trials)

**Food stimulus category**		**N food items**	**Tastiness**	**Healthiness**	**Familiarity**	**Liking**	**Calories (kcal)**	**Fat (g)**	**Sugar (g)**	**Salt (g)**	**Total sugars (inc. sugar)**	**Price per 100 g/mL**
conflict food images	tasty & unhealthy	200	5.4	3.0	3.7	4.3	375	19.4	29.5	0.7	40.9	1.49
		152	5.4	2.9	3.7	4.3	375	18.0	30.0	0.7	43.6	1.12
	untasty & healthy	200	3.7	4.8	3.1	3.4	151	4.9	6.5	0.5	21.0	1.96
		152	3.8	4.9	3.2	3.5	142	4.5	5.6	0.7	19.9	2.05
non-conflict food images	tasty & healthy	200	5.1	5.2	4.0	4.6	100	1.6	11.6	0.2	17.9	1.27
		152	5.0	5.2	3.9	4.6	101	1.2	11.9	0.2	18.2	1.14
	untasty & unhealthy	200	2.7	2.6	2.7	2.7	248	13.0	8.2	1.7	22.1	0.96
		152	2.7	2.5	2.7	2.7	236	12.2	7.0	1.3	19.5	0.91

SI Table 18. Seeds for parameter estimations

Inits	μ_{α}	$\boldsymbol{\sigma}_{\alpha}$	μ_{θ}	$\boldsymbol{\sigma}_{\theta}$	$\mu_{whealth}$	$\boldsymbol{\sigma}_{whealth}$	μ_{wtaste}	$\boldsymbol{\sigma}_{wtaste}$	μ_{bias}	bias. kappa	RNG	RNG. seed
1	0.5	0.05	0.1	0.05	0.3	0.05	0.01	0.05	0.4	1	Super-Duper	99999
2	0.3	0.05	0.2	0.05	0.3	0.05	0.1	0.05	0.5	1	Wichmann-Hill	1234
3	0.2	0.05	0.15	0.05	0.5	0.05	0.3	0.05	0.6	1	Mersenne-Twister	6666

μ – seed for the hyper-condition parameter mean
$\boldsymbol{\sigma}$ – seed for the hyper-condition parameter precision or error term
bias.kappa – seed weight of the shape and rate of the gamma prior distribution for the participant level bias parameter

SI Table 19. Parameter recovery. Pearson's correlation coefficients between generating and recovered parameters

Parameters	ALL N = 255
$w_{tastecp} - w_{healthcp}$	0.94, $p < 0.001$, 95% CI [0.92 – 0.95], BF_{10} = 6.4e+117
$bias_{cp}$	0.97, $p < 0.001$, 95% CI [0.96 – 0.97], BF_{10} = 6.4e+156
Non-decision time, θ_{cp}	0.99, $p < 0.001$, 95% CI [0.99 – 0.99], BF_{10} = 2.8e+234
$w_{tastecp}$	0.95, $p < 0.001$, 95% CI [0.94 – 0.96], BF_{10} = 6.9e+129
$w_{healthcp}$	0.91, $p < 0.001$, 95% CI [0.88 – 0.93], BF_{10} = 5.5e+92

SI Table 20. Average performance and reaction times during the MSIT (N = 53)

	Session 1		Session 2	
	**Decreased**	**Increased**	**Decreased**	**Increased**
Performance congruent	97.61 ± 1.45	98.57 ± 0.48	92.17 ± 4.49	97.92 ± 0.68
Performance incongruent	86.11 ± 3.23	90.70 ± 1.90	82.75 ± 5.04	90.63 ± 1.84
$\Delta_{Performance}$	11.50 ± 3.00	7.87 ± 1.70	9.42 ± 3.16	7.29 ±1.87
RT congruent	0.57 ± 0.03	0.54 ± 0.02	0.58 ± 0.03	0.56 ± 0.02
RT incongruent	0.89 ± 0.03	0.88 ± 0.03	0.89 ± 0.03	0.85 ± 0.02
Δ_{RT}	0.33 ± 0.02	0.34 ± 0.02	0.31 ± 0.02	0.30 ± 0.02

$\Delta_{Performance}$ – difference in performance between congruent trials and incongruent trials; RT – reaction time ; Δ_{RT} – difference in reaction times between congruent trials and incongruent trials. Source data are provided as a Source Data file.

SI Table 21. Whole brain activation for the contrast between incongruent > congruent MSIT conditions (N = 53).

Region	Brodmann Area	Side	Size	x	y	z	Peak z-score
V1	18	L	25142	-26	-90	-4	Inf
SMA	6	L	5164	-26	0	50	Inf
mPFC/IFG	9	L		-40	4	30	Inf
**dACC***	**32**	**L**		**-4**	**10**	**52**	**7.49**
IFG	6/9	R	782	44	6	28	7.04
Thalamus		L	965	-16	-16	14	6.71
**ant Insula***	**13**	**R**	**354**	**32**	**22**	**4**	**6.49**
	13	L	232	-30	20	6	6.61
**dlPFC/MFG*#**	**46**	**R**	**303**	**40**	**42**	**26**	**5.50**
	46	L	288	-44	38	26	6.11
Cerebellum		L	70	-24	-66	-48	6.03

V1 – primary visual cortex; SMA – supplementary motor area; IFG – inferior frontal gyrus; mPFC – medial prefrontal cortex; IFG – inferior frontal gyrus; dACC – dorsal anterior cingulate cortex; ant Insula – anterior insula; dlPFC – dorsolateral prefrontal cortex; MFG – medial frontal gyrus. Values were taken at a pFWE < 0.05 (family-wise error corrected based on peak height and cluster). *Regions of interest for the mediation analyses survived small volume correction using the main ROIs reported for the MSIT task by Bush et al. 2003[2]: dACC [3 10 47], Insula [32 23 10], dlPFC [47 33 27]. # dlPFC activation used for small volume correction of the PPI analysis.

SI Table 22. Whole brain activation for the contrast between congruent > incongruent MSIT conditions (N = 53).

Region	Brodmann Area	Side	Size	x	y	z	Peak z-score
PPC	39	L	1243	-46	-74	32	Inf
	39	R	976	46	-70	36	7.70
PCC	23/31	L	2637	-6	-56	14	7.14
vmPFC	10	L	5474	-2	58	-2	6.89
OFC/vlPFC	47	L	213	-38	34	-12	5.80
NAcc		R	30	8	8	-12	4.85
post Insula	13	R	200	36	-14	18	6.71
STG	21/22	L	513	-60	-14	-14	6.63
		R	323	54	-6	-20	6.19
Hippocampus		R	130	26	-18	-20	6.30
		L	307	-26	-20	-18	5.97
Fusiform	37	R	44	26	-36	-14	5.06
MTG	21	L	108	-56	-40	-4	5.00
Cerebellum		R	64	40	-74	-42	5.32
S1	1	R	43	52	-8	10	5.06

PPC – posterior parietal cortex; PCC – posterior cingulate cortex; vmPFC – ventromedial prefrontal cortex; OFC – orbitofrontal cortex; vlPFC – ventrolateral prefrontal cortex; NAcc – nucleus accumbens; post Insula – posterior insula; STG – superior temporal gyrus; MTG – middle temporal gyrus; S1 – primary somatosensory cortex. All values survived a pFWE < 0.05 (family-wise error corrected based on peak height).

9. Supplementary References

1. Krebs-Smith, S. M. *et al.* Update of the Healthy Eating Index: HEI-2015. *Journal of the Academy of Nutrition and Dietetics* **118**, 1591–1602 (2018).
2. Bush, G., Shin, L. M., Holmes, J., Rosen, B. R. & Vogt, B. A. The Multi-Source Interference Task: validation study with fMRI in individual subjects. *Mol Psychiatry* **8**, 60–70 (2003).
3. Bush, G. & Shin, L. M. The Multi-Source Interference Task: an fMRI task that reliably activates the cingulo-frontal-parietal cognitive/attention network. *Nat Protoc* **1**, 308–313 (2006).
4. Deng, Y., Wang, X., Wang, Y. & Zhou, C. Neural correlates of interference resolution in the multi-source interference task: a meta-analysis of functional neuroimaging studies. *Behav Brain Funct* **14**, 8 (2018).
