# Supplementary material for: Mapping expectancy-based appetitive placebo effects onto the brain in women
Source: Nat Commun. 2024 Jan 4;15:248. doi: 10.1038/s41467-023-44569-1 (PMC10764825; doi:10.1038/s41467-023-44569-1)
Supplement: Supplementary file 3 — Reporting Summary [file 41467_2023_44569_MOESM3_ESM.pdf]

nature portfolio

Corresponding author(s): Liane Schmidt

Last updated by author(s): 11/08/2023

Reporting Summary

Nature Portfolio wishes to improve the reproducibility of the work that we publish. This form provides structure for consistency and transparency in reporting. For further information on Nature Portfolio policies, see our Editorial Policies and the Editorial Policy Checklist.

Statistics

For all statistical analyses, confirm that the following items are present in the figure legend, table legend, main text, or Methods section.

n/a	Confirmed	
☐	☒	The exact sample size (*n*) for each experimental group/condition, given as a discrete number and unit of measurement
☐	☒	A statement on whether measurements were taken from distinct samples or whether the same sample was measured repeatedly
☐	☒	The statistical test(s) used AND whether they are one- or two-sided<br>*Only common tests should be described solely by name; describe more complex techniques in the Methods section.*
☐	☒	A description of all covariates tested
☐	☒	A description of any assumptions or corrections, such as tests of normality and adjustment for multiple comparisons
☐	☒	A full description of the statistical parameters including central tendency (e.g. means) or other basic estimates (e.g. regression coefficient) AND variation (e.g. standard deviation) or associated estimates of uncertainty (e.g. confidence intervals)
☐	☒	For null hypothesis testing, the test statistic (e.g. *F*, *t*, *r*) with confidence intervals, effect sizes, degrees of freedom and *P* value noted<br>*Give P values as exact values whenever suitable.*
☐	☒	For Bayesian analysis, information on the choice of priors and Markov chain Monte Carlo settings
☒	☐	For hierarchical and complex designs, identification of the appropriate level for tests and full reporting of outcomes
☐	☒	Estimates of effect sizes (e.g. Cohen's *d*, Pearson's *r*), indicating how they were calculated

Our web collection on statistics for biologists contains articles on many of the points above.

Software and code

Policy information about availability of computer code

Data collection	Matlab 2018b (Mathworks) and Psychtoolbox
Data analysis	Matlab 2018b Statistical and Machine learning toolbox, JASP v0.16.2(Intel), RStudio v1.3.1093 (2009–2020 RStudio, PBC)

For manuscripts utilizing custom algorithms or software that are central to the research but not yet described in published literature, software must be made available to editors and reviewers. We strongly encourage code deposition in a community repository (e.g. GitHub). See the Nature Portfolio guidelines for submitting code & software for further information.

Data

Policy information about availability of data

All manuscripts must include a data availability statement. This statement should provide the following information, where applicable:

- Accession codes, unique identifiers, or web links for publicly available datasets
- A description of any restrictions on data availability
- For clinical datasets or third party data, please ensure that the statement adheres to our policy

The source data generated in this study have been deposited in the Open Science Framework (OSF) repository [https://osf.io/7j4qs/?view_only=d5c0886514d740c293ebdc14524f37a6]. The fMRI data files are available under restricted access for limited OSF storage space reasons; access can be obtained by contacting the corresponding author. The raw behavioral data are protected and are not available due to data privacy laws. The metadata generated in this study are provided in the Supplementary Information/Source Data file.

Research involving human participants, their data, or biological material

Policy information about studies with human participants or human data. See also policy information about sex, gender (identity/presentation), and sexual orientation and race, ethnicity and racism.

Reporting on sex and gender	Findings apply to self-identified female participants only. Gender/sex were determined based on self-reporting, and analyses were restricted to female participants to control for sex/gender-based differences in dietary self-control and placebo effects (Rolls et al. 1991, Olson et al. 2021, Colloca et al. 2016, Theysohn et al. 2014, Davy et al. 2006, Marino et al. 2011, Frank et al. 2010).
Reporting on race, ethnicity, or other socially relevant groupings	The reporting of race, ethinicity and socially relevant groupings is prohibited by the law for biomedical research in France (Data Protection Act No. 78-17, Article 6 created by the CNIL 1978 and in action since 2019). This information was therefore not collected.
Population characteristics	age=34.9 +- 1 years, right-handed, corrected to normal vision, no past history of substance abuse, neurological or psychiatric disorder, absence of metallic devices, no medication
Recruitment	Participants were recruited via public advertissment (f;ex., on the CNRS risc website) to reach a divers population of potential participants in the Paris area. Participants were recruited on the basis of a-priori established inclusion criteria (i.e., normal to corrected-to-normal vision, no history of substance abuse or any neurological or psychiatric disorders, and no medication). Participants of the fMRI sample were additionally screened for the absence of metallic devices and right-handedness. The authors are not aware of selection biases or other biases that might have emerged from this way of recruiting, and how they might have impacted results.
Ethics oversight	Comité de Protection des Personnes (CPP) Ile de France VI N°1204, EST III n°19.12.05

Note that full information on the approval of the study protocol must also be provided in the manuscript.

Field-specific reporting

Please select the one below that is the best fit for your research. If you are not sure, read the appropriate sections before making your selection.

☐ Life sciences ☒ Behavioural & social sciences ☐ Ecological, evolutionary & environmental sciences

For a reference copy of the document with all sections, see nature.com/documents/nr-reporting-summary-flat.pdf

Behavioural & social sciences study design

All studies must disclose on these points even when the disclosure is negative.

Study description	The study was designed as an interventional study with minimal risks for the human participant. The design generated quantitative data that was collected in person.
Research sample	Mean age = 34 +-1 years, all female. The sample is representative for female participants of this age group. The rational for a solely female participant sample was to control for sex/gender-based differences in dietary self-control and placebo effects (Rolls et al. 1991, Olson et al. 2021, Colloca et al. 2016, Theysohn et al. 2014, Davy et al. 2006, Marino et al. 2011, Frank et al. 2010).
Sampling strategy	The sampling strategy for the two hunger suggestion groups was random.<br>The sample size for fMRI was determined (1) by recommendations from the field (Woo et al. NeuroImage 2014), (2) reality of participant recruitment for an in-person fMRI experiment during the COVID-19 pandemic, and (3) to meet the estimations from a power analysis to replicate the behavioral effect of suggestion on hunger ratings (Cohen's d=0.7) observed in the behavioral pilot sample (n=115) with a power between 0.7 (two-tailed, n=31 per group) and 0.8 (one-tailed, n=30 per group) at a threshold of p<0.05.
Data collection	Computer-based data collection, pilot behavioral study participants were performing the decision-making task and questionnaire ratings alone. The experimenter was not present in the testing room. Participants of the fMRI study performed the task inside an fMRI scanner. The experimenters were not blind to the study design.
Timing	Pilot data was collected between November 2017 and September 2018, FMRI data was collected between June 2020 and June 2021. Behavioral control group was collected from Mai to July 2023.
Data exclusions	Exclusion criteria were a baseline hunger rating < 2 (no hunger), pregnancy, claustrophobia, permanent make-up or metallic implants that were not reported at the time of recruitment, and technical problems with the fMRI scanner. Based on these exclusion criteria, 23 participants were excluded from the data analysis due to problems with the fMRI scanner (n = 1) and not being hungry after overnight fasting at baseline (n = 5 in the decreased-hunger suggestion group, n = 10 in the increased-hunger suggestion group, and n=7 in the control group).
Non-participation	Two participants dropped out after signing the consent form due to fMRI contra-indications that were not detected at time of recruitment. No behavioral and fMRI data was obtained from these participants.
Randomization	The probability of being assigned to one of the two hunger suggestion arms was set to p = 0.5 and remained the same for the

duration of the study. Randomization was performed before participants were enrolled using standard permutation algorithms implemented in MATLAB. The algorithm drew one of the two integers 1 and 2. If the integer was '1', the participant was assigned to the suggestion group 1 (decreased-hunger suggestion). If it was '2', the participant was assigned to suggestion group 2 (increased-hunger suggestion). To ensure an equal number of participants in each suggestion group the permutation was repeated 63 times for the behavioral pilots and 31 times for the fMRI participants.
Note, the initial pre-registration of the study did not foresee a control group, which was added a-posteriori. The randomization was therefore conducted on the two hunger suggestion groups.

Reporting for specific materials, systems and methods

We require information from authors about some types of materials, experimental systems and methods used in many studies. Here, indicate whether each material, system or method listed is relevant to your study. If you are not sure if a list item applies to your research, read the appropriate section before selecting a response.

Materials & experimental systems

n/a	Involved in the study
☒	☐ Antibodies
☒	☐ Eukaryotic cell lines
☒	☐ Palaeontology and archaeology
☒	☐ Animals and other organisms
☒	☐ Clinical data
☒	☐ Dual use research of concern
☒	☐ Plants

Methods

n/a	Involved in the study
☒	☐ ChIP-seq
☒	☐ Flow cytometry
☐	☒ MRI-based neuroimaging

Magnetic resonance imaging

Experimental design

Design type: Event-related task design, two groups of participants

Design specifications: 152 trials, each trial lasted between 8.5 seconds (minimal) and 10.5 seconds (maximal)

Behavioral performance measures: 4-point Lickert ratings of food stimulus value (i.e., How much do you want to eat this food?, 1-strong no, 2-no, 3-yes, 4-strong yes). Reaction times in seconds.

Acquisition

Imaging type(s): functional and structural

Field strength: 3 T

Sequence & imaging parameters: T2*-weighted multi-echoplanar images (mEPI) . Each volume comprised 48 axial slices collected in an interleaved manner. To cover the entire brain, the acquisition sequence involved the following parameters: echo times of 14.8 ms, 33.4 ms, and 52 ms; FOV = 192 mm; voxel size = 3 x 3 mm; slice thickness = 3 mm; flip angle = 68°; and TR = 1.25s.

Area of acquisition: Whole brain

Diffusion MRI ☐ Used ☒ Not used

Preprocessing

Preprocessing software: Statistical Parametric Mapping software (SPM 12,FIL)

Normalization: Normalization to the Montreal Neurological Institute (MNI) space using the same transformation as for the participant's anatomical image

Normalization template: specific subject space from anatomical image.

Noise and artifact removal: The 6 motion parameters obtained from the realignment preprocessing step were included in all firstlevel statistical analyses as covariates of non-interest to control for head movement.

Volume censoring: NaN

Statistical modeling & inference

Model type and settings: mass univariate. Multilevel general linear models with random effects group comparisons of individual beta images for choice-onset and stimulus value using two-sampled t-tests.

Effect(s) tested: difference between groups in beta images for choice onset and the parametric modulator stimulus value

Specify type of analysis: ☐ Whole brain ☐ ROI-based ☐ Both

Statistic type for inference (See Eklund et al. 2016): Whole brain analyses: cluster-and peak-level pFWE<0.05. Small volume correction analyses: primary (initial) uncorrected whole brain threshold of p<0.001, a 10 mm radius sphere centered around independantly, functionally and anatomically defined regions's of interest, and inferences drawn at cluster and peak level pFWE)<0.05.

Correction: family wise error correction (FWE) on the cluster and peak level

Models & analysis

n/a	Involved in the study
☐	☒ Functional and/or effective connectivity
☒	☐ Graph analysis
☒	☐ Multivariate modeling or predictive analysis

Functional and/or effective connectivity: Psycho-physiological analysis evaluating the covariance of the seed region: vmPFC to the dlPFC ROI at time of choice versus fixation. Beta estimates for this PPI contrats were correlated to drift weights obtained for healthiness and tastiness information from a DDM model using Pearson's r. ROI based mediation analyses.
